# Secondary Hyperparathyroidism in Chronic Kidney Disease: Pathophysiology and Management

**DOI:** 10.7759/cureus.16388

**Published:** 2021-07-14

**Authors:** Elmukhtar Habas, Mohsen Eledrisi, Fahmi Khan, Abdel-Naser Y Elzouki

**Affiliations:** 1 Internal Medicine, Hamad Medical Corporation, Doha, QAT; 2 Internal Medicine, Hamad General Hospital, Doha, QAT

**Keywords:** secondary hyperparathyroidism, hypocalcemia, hyperphosphatemia, chronic kidney disease, hungry bone syndrome

## Abstract

Serum calcium concentration is the main determinant of parathyroid hormone (PTH) release. Defect in the activation of vitamin D in the kidneys due to chronic kidney disease (CKD) leads to hypocalcemia and hyperphosphatemia, resulting in a compensatory increase in parathyroid gland cellularity and parathyroid hormone production and causing secondary hyperparathyroidism (SHP). Correction and maintenance of normal serum calcium and phosphate are essential to preventing SHP, hungry bone disease, cardiovascular events, and anemia development. Understanding the pathophysiology of PTH and possible therapeutic agents can reduce the development and associated complications of SHP in patients with CKD. Medical interventions to control serum calcium, phosphate, and PTH such as vitamin D analogs, calcium receptor blockers, and parathyroidectomy are needed in some CKD patients. In this review, we discuss the pathophysiology, clinical presentation, and management of SHP in CKD patients.

## Introduction and background

Parathyroid hormone (PTH) is a polypeptide protein released by the parathyroid gland [1]. PTH plays an essential role in bone mineralization and calcium and phosphate homeostasis by enhancing tubular calcium reabsorption in the kidneys, calcium absorption in the gastrointestinal tract, calcium mobilization from the bones, and phosphate excretion by the kidneys. The metabolically active form of vitamin D, 1,25-dihydroxycholecalciferol (1,25(OH)_2_D) stimulates intestinal calcium absorption and bone calcium mobilization. PTH, vitamin D, fibroblast growth factor-23 (FGF-23), phosphate, and calcium ion regulate serum calcium hemostasis [2]. Serum phosphate levels are high in primary and secondary hyperparathyroidism and low in primary hyperparathyroidism due to increased renal phosphate excretion.

## Review

Physiology of parathyroid hormone

**Figure 1:**
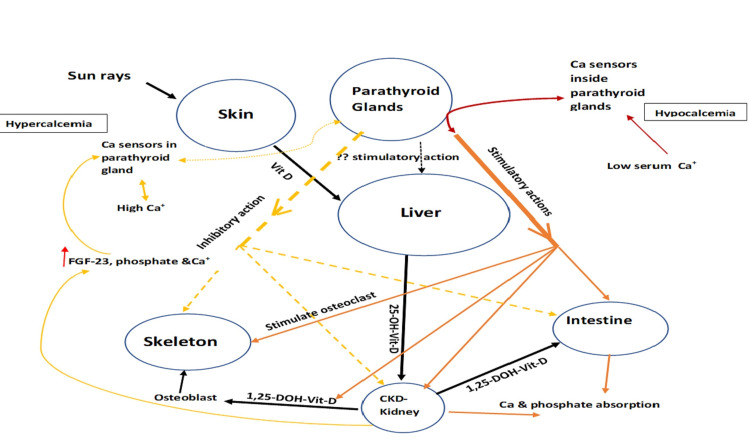
Physiology of PTH. 1,25-DOH-Vit-D: 1,25 dihydroxy hydroxycholecalciferol; 25-OH-Vit-D: 25 monohydroxy hydroxycholecalciferol; CKD: chronic kidney disease; FGF: fibroblast factor; PTH: parathyroid hormone; Vit D: vitamin D

The main controller of PTH synthesis and secretion is the extracellular free (ionized) calcium [3]. Inorganic serum phosphate has contributory effects on PTH production and release by the parathyroid glands. The parathyroid glands have calcium sensor receptors (CaSR) that sense the changes in plasma calcium concentrations and regulate PTH secretion [4]. In addition, PTH synthesis and secretion are affected by 1,25(OH)_2_D formed by the conversion of 25 hydroxyvitamin D under the influence of the 1-alpha hydroxylase enzyme in the renal proximal tubular cells. 1,25(OH)_2_D binds to the parathyroid vitamin D receptor (VDR) which affects the synthesis of PTH-messenger RNA (PTH-mRNA) [5]. FGF-23 and osteocyte and osteoblast-derived phosphaturic hormone [6] have been shown to decrease PTH synthesis and secretion [7]. Figure 1 shows the physiology of PTH.

Pathophysiology of secondary hyperparathyroidism

**Figure 2:**
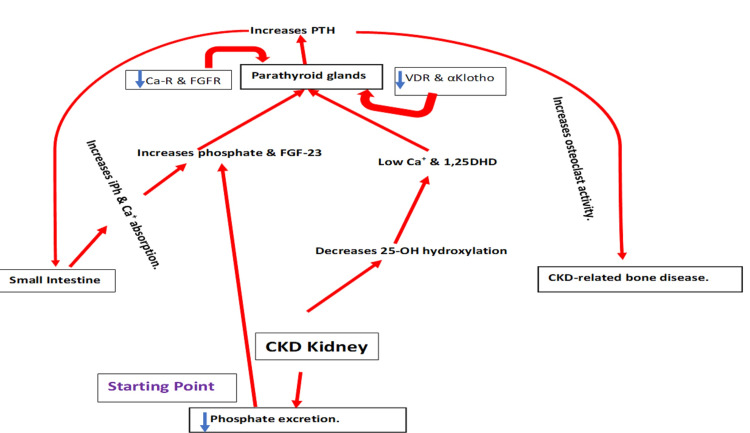
Pathophysiology of SHP. 1,25DHD: 1,25 dihydroxy vitamin D; Ca-R: calcium receptor; CKD: chronic kidney disease; FGF-23: fibroblast growth factor-23; FGFR: fibroblast growth factor receptor; iPh: inorganic phosphate; PTH: parathyroid hormone; SHP: secondary hyperparathyroidism; VDR: vitamin D receptor

Prolonged low serum calcium levels trigger PTH secretion, leading to parathyroid cell proliferation and hyperplasia [8]. In addition, serum phosphate concentration affects PTH synthesis, secretion, parathyroid cell proliferation, vitamin D activation, and FGF-23 formation [9,10]. Moreover, PTH secretion has been shown to be dependent on phosphate concentration in bovine and rat parathyroid tissue [11,12]. In normal and uremic experimental models, high-phosphorus diets increase serum PTH levels [13]. Although the effect of serum phosphate level on serum PTH is not sufficiently clear, recent data have speculated that in addition to sensing serum calcium changes, CaSR can sense the changes in serum phosphate level [14]. Activation of 25-hydroxy vitamin D to 1,25(OH)_2_D is controlled by PTH in the kidneys. 1,25(OH)_2_D employs a negative feedback effect to downregulate PTH expression. FGF-23 expression in bone osteocytes is regulated by phosphate, 1,25(OH)_2_D, and PTH. Furthermore, increased FGF-23 acts on the parathyroid fibroblast growth factor receptor (FGFR1)-αKlotho receptor complex to downregulate PTH expression [15,16]. Figure 2 shows the pathophysiology of SHP.

In chronic kidney disease (CKD) patients, serum phosphate increases due to reduced phosphate secretion by the renal tubules, promoting the risk of development of SHP [17]. Furthermore, high serum phosphate has been reported to inhibit proximal renal tubule hydroxylase enzyme, complicating and further increasing the risk of hyperphosphatemia in CKD patients [18]. A progressive decline in glomerular filtration rate (GFR) in CKD leads to a reduction in the levels of 1,25(OH)_2_D. The prolonged CKD-associated hypocalcemia and hyperphosphatemia drive the parathyroid gland to secrete more PTH to normalize the serum calcium and phosphate levels, leading to SHP. Nevertheless, this theory is not widely accepted because serum 1,25(OH)_2_D decreases during the early stages of CKD even before hypocalcemia and hyperphosphatemia occur. In the last decade, the discovery and characterization of the *FGF-23* gene located on chromosome 12 have helped in understanding the early phases of the pathogenesis of SHP [19]. 1,25(OH)_2_D, phosphate, PTH, and calcium promote the synthesis and release of this protein by an unknown mechanism, although it is assumed that PTH induces FGF-23 transcription by triggering the bone-parathyroid-endocrine loop [20].

The membrane-bound αKlotho and FGF-23 along with their coreceptor induce phosphaturia through suppression of the sodium phosphate cotransporter [21]. αKlotho is produced by the kidneys, is considered to be an early biomarker for CKD, and may have extra autonomous anticalciuric and phosphaturic action, independent of FGF-23 [22]. In CKD, assembly of both membrane-bound and αKlotho leads to an increase in the serum levels of FGF-23 due to decreased FGF-23 clearance, leading to phosphate retention. The resistance to the phosphaturic action of FGF-23 is due to αKlotho deficiency, which becomes apparent with phosphate retention as CKD progresses.

αKlotho was originally recognized as an aging feature suppressor [23]. It is a single-pass transmembrane protein that acts as an FGF-23 coreceptor [24]. αKlotho is widely expressed, although it is predominantly higher in the kidneys [25]. As shown in an animal study, αKlotho deficiency leads to a premature aging syndrome associated with ectopic soft tissue calcification [23]. Overexpression of αKlotho reduces soft tissue calcification, indicating that αKlotho has an inhibitory effect on ectopic calcification [23].

FGF-23 levels increase as CKD progresses, leading to a “trade-off mechanism” between normalizing serum phosphate concentrations and activation of 1,25(OH)_2_D, which may cause severe SHP [26]. In the late stages of CKD, hyperphosphatemia, decreased production of 1,25(OH)_2_D, and hypocalcemia promote PTH-mRNA transcription and PTH formation, leading to SHP.

Persistent hypocalcemia leading to hyperplasia of parathyroid cells and downregulation of VDRs and CaSR in the parathyroid glands increase PTH secretion [27]. It has been postulated that FGF-23 may decrease PTH secretion via the αKlotho-FGFR1 complex [20]. In advanced SHP, the Klotho-FGFR1 complex expression in parathyroid cells is impaired [28], leading to an increase in the inhibitory effect of FGF-23 resistance to PTH secretion. Through activation of the mitogen‑activated protein kinase pathway, FGF-23 acts on the receptor complex in the parathyroid glands to reduce PTH gene expression and secretion. Both FGF-23 and PTH are elevated in CKD, suggesting that the parathyroid glands are resistant to FGF-23 [29].

It has been reported that 30‑50% of patients with stage 5 CKD have PTH levels of >300 pg/mL [30]. An earlier response that becomes maladaptive with CKD progression is a clinical syndrome termed chronic kidney disease‑mineral and bone disorder (CKD‑MBD) [31]. CKD-MBD is a disorder of mineral and bone metabolism that occurs due to an imbalance between PTH, serum calcium, phosphorus, and vitamin D metabolism. CKD-MBD causes bone abnormalities that affect turnover, volume, mineralization, vascular, linear growth, and density, and soft tissue calcification. CKD-MBD starts usually in stage 2 CKD [32], and is associated with vascular calcification, osteodystrophy, αKlotho loss, and increased FGF-23 secretion [32]. Associated skeletal manifestations have been shown to improve after controlling the serum PTH concentration [33], likely by mitigating the bone high turnover process (i.e., osteitis fibrosa) and improving the bone mineral density [34]; however, the extraskeletal complications did not improve [35].

Clinical features of secondary hyperparathyroidism in chronic kidney disease

Fragility fractures occur two to four times more frequently in patients with CKD-associated SHP compared to their age- and sex-matched healthy individuals. The risks of bone fractures are in the range of 15.0%, 20.5%, 24.2%, 31.2%, and 46.3% per 1,000 person-years for CKD stages 1, 2, 3a, 3b, and 4, respectively [36]. Skeletal fracture risk is up to five times more in individuals with an estimated glomerular filtration rate (eGFR) of <15 mL/minute versus >60 mL/minute per 1.73 m^2^. Furthermore, fracture incidence is significantly higher in hemodialysis patients than in the general population, associated with a 3.7-fold increase in the unadjusted relative risk of death [37]. Other causes of reduced bone fragility and bone fracture such as metabolic acidosis, anemia, hypogonadism, inflammation, β2 microglobulin associated with amyloidosis, vitamin D deficiency, bone formation inhibition secondary to osteoblast cell inhibition, along with that are not uncommon in CKD should be considered [34].

SHP causes vascular calcification of the coronary vessels and other peripheral vessels, causing ischemic cardiovascular events and heart failure. Furthermore, higher PTH in SHP increases sympathetic drive and endothelial stress [38]. Intimal and medial layer vascular calcification have been identified in CKD patients. Intimal calcification induced by CKD takes the form of atherosclerosis plaque calcification by increased osteoclastic in the neointimal layer. This type of calcification is originally related to small muscle cells and mesenchymal cells [39]. Mid-vascular layer calcification has also been correlated with chondro-osseous smooth vascular muscle cell transition [40]. High plasma DKK1 protein, sclerostin, bone morphogenetic protein 9 (BMP-9) [41], and activin have been observed in CKD patients [42].

Mild and moderate CKD increases coronary atherosclerotic disease risk by 87% [43]. The causes of an increased risk of cardiovascular disease are linked to CKD-MBD [44]. Three new cardiovascular risk factors have been identified within the CKD-MBD, namely, hyperphosphatemia, vascular calcification, and increased FGF-23 levels [45]. An animal study claimed that continuous infusion of PTH in CKD rats led to the development of hypercalcemia and severe calcification of the aortal medial layer [46]. On the contrary, intermittent teriparatide (PTH analog) in diabetic male mice prevented vascular calcification and aortic osteogenic transformation [47]. Though both studies could not explain the identical effects of PTH, both high serum calcium and phosphate and the high value of their product are recognized risk predictors of vascular calcification in patients with CKD. Moreover, SHP is directly related to serum alkaline phosphatase which is a good predictor of coronary artery calcification in CKD and hemodialysis patients [48]. Therefore, vascular calcification in SHP patients appears to be mediated by alkaline phosphatase, and this biological theory appears sensible because survival in these patients improves after parathyroidectomy.

Calciphylaxis or calcific uremic arteriolopathy is uncommon in patients with CKD and manifests as skin necrosis and severe ulcers that are difficult to heal. High PTH levels correlate with the occurrence of calciphylaxis, and parathyroidectomy is associated with a decreased mortality in this setting [49]. Parathyroidectomy does not result in improvement of vascular and soft tissue calcification [50].

SHP has been associated with resistance to the effects of erythropoietin therapy in patients with CKD-associated anemia; this is defined as failure to normalize hemoglobin after four to six months of erythropoietin therapy in the absence of iron deficiency [51]. The mechanisms by which SHP may cause anemia or reduce the bone marrow response to erythropoiesis are not clearly understood. High PTH levels can affect red blood cell production either directly via the toxic effect of PTH on bone marrow erythroid progenitors and increased hemolysis or indirectly through promoting bone marrow fibrosis [52]. This theory is supported by an improvement of anemia and reduction of bone marrow fibrosis following parathyroidectomy [53].

Treatment of Secondary Hyperparathyroidism

Controlling serum levels of PTH, phosphorus, calcium, and 25-hydroxy vitamin-D improves morbidity and mortality in patients with CKD [54].

Treatment of hyperphosphatemia

Controlling serum phosphorus is an essential step in the management of SHP. In CKD stages 2, 3, and 4, dietary phosphorus restriction is enough to maintain normal serum phosphorus levels [55]. Protein restriction is required to prevent renal function deterioration; however, daily protein requirement should be prescribed, while severe restriction may lead to malnutrition in CKD patients. Furthermore, patients’ appetite decreases as kidney function worsens, increasing the risk of malnutrition. Therefore, vegetable protein is advisable more than anima protein, and processed food containing conservatives, high potassium, and salt should be avoided in patients with CKD.

As hyperphosphatemia becomes persistent and severe in patients with CKD, phosphate binders must be started. Phosphate binders are classified into calcium-based and noncalcium binders. Both types have almost the same effect in controlling the serum phosphate and serum PTH concentration in CKD-associated SHP. Calcium-containing binders include calcium acetate and calcium carbonate. Major noncalcium-containing binders are sevelamer and lanthanum. Other agents include ferric citrate and sucroferric oxyhydroxide are used. The main side effect of calcium-containing binder is hypercalcemia [56].

Sevelamer is a resin noncalcium phosphate binder used to treat hyperphosphatemia. A meta-analysis study concluded that sevelamer reduced mortality more than other calcium phosphate binders [57]. Lanthanum carbonate is another noncalcium-based phosphate binder that prevents the intestinal absorption of phosphate. In addition to nausea, vomiting, and constipation, it may cause bowel obstruction or perforation. Lanthanum carbonate is primarily metabolized by the liver and is eliminated in bile. Although high serum levels of lanthanum carbonate have little effect on the bones, it may cause bone discomfort. Furthermore, lanthanum carbonate can produce elevated liver enzymes; however, this is usually not serious. Although lanthanum carbonate does not cross the blood-brain barrier, it can cause convulsions. Despite the mentioned and other side effects of lanthanum carbonate, they have no clinical implications if the patient and the treating physician are aware of them and intervene quickly if any of them are noted.

It has been recently reported that adding enteral sodium-phosphate (NPT2b) transporter blockades to nicotinamide plus lanthanum carbonate, nicotinamide plus placebo, or lanthanum carbonate plus placebo did not lead to any significant difference in hyperphosphatemia and serum FGF-3 level [58]. More studies are required to investigate their role in controlling hyperphosphatemia. Inhibition of enteral sodium/hydrogen exchanger-3 by tenapanor that was approved by the FDA in 2019 decreases phosphate absorption in patients on hemodialysis. However, the adverse effects of tenapanor such as increased stool sodium and water loss limited its widespread use [59].

Vitamin D analogs

Calcitriol is a vitamin D receptor activator (VDRAs) for the treatment of SHP to control hyperphosphatemia in patients with CKD [60]. It reduces bone turnover and prevents osteitis fibrosa in patients on hemodialysis [61]. Calcitriol is the usual agent to regulate serum PTH in SHP patients [62]. The possibility of hyperphosphatemia and hypercalcemia increases with high doses of calcitriol, which can increase the risk of morbidity and mortality in CKD-induced SHP.

Gene transcription regulation, inhibition of PTH-mRNA synthesis, and specific VDR-modifying agents have an inhibitory effect on PTH synthesis. In severe cases of SHP, there is a reduction of CaSR and VDRAs in the parathyroid glands [63]. Furthermore, VDRAs in advanced SHP do not effectively inhibit PTH secretion [64].

Selective VDRA vitamin D analogs such as paricalcitol (19‑nor‑1,25‑dihydroxy vitamin D2) and maxacalcitol (22‑oxa‑1,25‑dihydroxy vitamin D3) can reduce PTH levels better than calciferol without increasing the serum calcium concentration; however, it can cause hypercalcemia in some SHP patients. Native vitamin D esters are effective, and can be still used to decrease plasma PTH during earlier stages of SHP [64].

Calcimimetics

Cinacalcet decreases serum calcium and PTH efficiently in CKD-associated SHP [65]. Cinacalcet acts as a calcimimetic by allosteric G protein-coupled receptor modulator to activate the calcium-sensing receptor. Long-term use of cinacalcet reduces and improves the high bone turnover rate in end-stage renal disease among regular hemodialysis patients with SHP [66]. Furthermore, cinacalcet reduces the risk of death due to cardiovascular complications of SHP [67]. Additionally, cinacalcet reduces the risk of bone fracture compared with the placebo group [68]. The main side effects of cinacalcet are nausea and vomiting. Etelcalcetide is another calcimimetic agent which reduces PTH levels in patients on hemodialysis more effectively than cinacalcet without causing nausea and vomiting [69]. Paricalcitol (a selective vitamin D analog) has a limited action on intestinal and bone VDRs, but it decreases serum PTH levels.

Parathyroidectomy

In chronic hemodialysis patients, parathyroidectomy is required to control hyperparathyroidism in 15% and 35% of patients after 10 years and 35 years from the onset of hemodialysis, respectively [70]. Symptoms such as itching, bone pain, bone fracture, quality of life, and mortality are improved in some patients after parathyroidectomy [70]. In addition to the operative complications, hypophosphatemia, hypocalcemia, and occurrence and progression of the hungry bone syndrome are the most severe and common complications due to sudden serum PTH reduction following parathyroidectomy [71]. The hungry bone syndrome occurs in 5-8% of patients with SHP after parathyroidectomy, and sometimes it may not be detected early, causing adynamic bone disease [72]. In hemodialysis patients, an adynamic bone disease noted following parathyroidectomy is associated with worsening of vascular calcification [73]. Post-parathyroidectomy hypoparathyroidism is not a common complication in SHP as in primary hyperparathyroidism.

Chemical ablation of parathyroid glands by percutaneous ethanol injection was conducted in 1985 as a treatment for SHP [74]. In 2003, a guideline included the use of ethanol to destroy the nodular parathyroid gland. Other hyperplasic glands are managed medically, and/or later can be treated by ethanol injection if medical treatment fails [75]. Furthermore, calcitriol percutaneous injection has been also used to destroy the hyperactive parathyroid gland instead of ethanol [76].

However, percutaneous ethanol and calcitriol are sometimes used for SHP patients who refuse or are not good candidates for surgical parathyroidectomy, and the long‑term control of PTH level is not always achievable.

SHP may persist following renal transplantation, particularly in patients who had nodular SHP before parathyroidectomy, leading to hypercalcemia [77], and renal graft failure due to vascular calcification risk [78]. Surgical parathyroidectomy should be considered in kidney transplant patients with persistent hyperparathyroidism, especially when it is associated with hypercalcemia. Cinacalcet can be used as a bridging agent in renal transplant patients who have persistent hypercalcemia before parathyroidectomy [79].

## Conclusions

The pathogenesis of SHP in patients with CKD has recently improved. The link between SPH and its consequences, including bone disease, vascular calcification, and anemia, requires more research. More therapeutic approaches for reducing the need for parathyroidectomy and its complications must be researched further in terms of their safety and efficacy.
